# The effect of milling time on the preparation of an aluminum matrix composite reinforced with magnetic nanoparticles

**DOI:** 10.1016/j.heliyon.2023.e16887

**Published:** 2023-06-01

**Authors:** Ayman Elsayd, Ahmed Y. Shash, Hisham Mattar, Per A. Löthman, Mohamed E. Mitwally

**Affiliations:** a Central Metallurgical Research and Development Institute, Cairo, Egypt; b Mechanical Design and Production Engineering Department, Faculty of Engineering, Cairo University, 12316, Giza, Egypt; c The German University in Cairo, Egypt; d German International University, Egypt; e Foviatech GmbH, Hamburg, Germany; f Kaiserslautern University of Applied Science, Zweibrücken, Germany

**Keywords:** Metal matrix composites, Aluminum matrix composites, Magnetite, Powder metallurgy, Ball milling

## Abstract

Powder metallurgy methods, particularly ball milling, are up-and-coming in tuning metal matrix composite (MMC) properties. This study uses ball milling at various milling times to create an aluminum matrix composite (AMC) reinforced with magnetite nanoparticles. The milling time was optimized to create an AMC with favorable mechanical and magnetic properties, and its effect on magnetism, microstructure, and hardness was studied.

The AMC displayed the highest magnetic saturation of 11.04 emu/g after 8 h of milling. After compaction and sintering, characterization of the final composite material using Energy Disperse Spectroscopy and X-ray diffraction (XRD) showed the presence of Al_2_O_3_ and Fe_3_Al phases leading to enhanced mechanical properties in terms of Vickers hardness that reached a value of 81 Hv corresponding to an increase of 270% compared to unreinforced aluminum.

## 1 Introduction

Powder Metallurgy (PM) has proven to be a reliable method of manufacturing net-shape components that allow for less machining and higher production rates, thus lowering total cost [1]. Furthermore, PM technology can refine microstructures compared to standard machining processes, resulting in superior mechanical and corrosion properties [2,3]. Furthermore, metal matrix composites (MMCs) made with the PM technique usually have improved mechanical, electrical, and thermal properties. Accordingly, MMCs are used in thermomechanical applications, high-performance mechanical structures, and applications requiring tailored magnetic and electrical properties [4,5].

Out of the different MMCs, aluminum matrix composites (AMCs) draw the attention of both industry and academia due to their direct use in aerospace, energetics, structural, renewable energy, and electronic/electric applications [6,7]. One reason for the high interest in AMCs is the abundance of aluminum in the earth's crust compared to other ferrous and non-ferrous metals [8]. Moreover, aluminum has several advantages compared to other metals, such as low density, high ductility, corrosion resistance, and high thermal conductivity [9]. On the other hand, it is inherently weaker than known ferrous alloys. For example, most aluminum alloys have a tensile strength of around 483 MPa [10], while ferrous alloys show a tensile strength of about 1000 MPa [10]. Furthermore, aluminum shows poor tribological performance in wear-prone applications [11].

These constraints can be solved by boosting aluminum's mechanical strength using particle ceramic reinforcing elements. Carbides, borides, and oxides are the most commonly used reinforcing materials [2,3,7]. Incorporating reinforcing materials improves the mechanical properties of AMCs while also enhancing electric and thermal properties, as in the case of Carbon Nanotubes or Graphene, and magnetic permeability, as in the case of Magnetite nanoparticles (MagNPs) [[2], [3], [12], [13], [14], [15], [16]].

One of the main iron ores and a relatively common mineral is Magnetite (Fe_3_O_4_). It is one of the most potent ferric magnetic minerals. It has the highest iron content (72.4%) and the highest magnetization saturation among iron oxides [16]. In its nano-form, it is highly versatile and is used in medical applications such as magnetic resonance imaging (MRI) and hyperthermia [17]. Magnetite nanoparticles (MagNPs) can also be used as catalysts, heavy metal adsorbents in water purification [18], and functional reinforcements in composites. Moreover, these composites can also be used in electromagnetic applications like high-frequency transformers, converter inductor cores, and electric motors, thanks to the increased magnetic permeability of MagNPs [19].

The inclusion of MagNPs as reinforcement in AMCs has been investigated in several studies focusing on the variation of properties as a function of MagNPs weight percentage (wt%). It was reported that as magnetite concentration increases, the mechanical properties are improved, mainly hardness and compression strength. The magnetic affinity of the composite increases as well, from a weak paramagnetic material with low magnetic susceptibility to a more substantial magnetic composite. The effects of various sintering techniques were studied, comparing the most common vacuum sintering technique to the microwave sintering technique claimed to produce better-sintered composites at lower time intervals. The milling and mixing parameters, however, were fixed. In addition, the milling time was not investigated as a varying parameter in the properties of the different AMCs [[20], [21], [22], [23], [24], [25], [26]].

As such, the current study aims to fill such gap in the literature and investigate the effect of the milling time as the optimizing milling parameter in fabricating MagNPs-reinforced AMCs. Furthermore, the mechanical and magnetic performance of the resulting composites were compared to similar composites reported in literature.

## 2 Experimental procedure

### 2.1 Materials

This work used aluminum powder of 99.9% purity as the matrix material (DOP ORGANIK KIMYA SAN.VE TIC. LTD.STL). The average particle size was 10 μm. For the reinforcing material, the MagNPs were prepared via a modified method introduced by Liang et al. [24]. In an inert atmosphere, 5.56 g of FeSO_4._7H_2_O and 11.60 g of FeCl_3_·6H_2_O were dissolved in deionized water.

The solution was heated to 80 °C, vigorously stirred, and 20 mL ammonium hydroxide was added. For 10 min, the suspension was vigorously stirred, then the mixture was left to cool to ambient temperature.

The black product (slurry) was extracted using a strong neodymium magnet and thoroughly washed with ethanol and deionized water in multiple consecutive cycles to remove excess ammonia and chemical residues. The resulting Fe_3_O_4_ nanoparticles were vacuum-dried for 12 h at 60 °C. The obtained powder was characterized using high-resolution transmission electron microscopy (TEM), X-ray diffraction (XRD), and vibratory sample magnetometry (VSM).

### 2.2 Composite preparation

The powder mixture was prepared for the milling process by mixing MagNPs with aluminum powder in a ratio of 1:9 by weight. The powder mixture was then processed in a planetary ball mill at 200 rpm, with steel balls in a ball-to-powder ratio of 4:1. The powder was milled for 2, 4, 6, and 8 h with the as-milled powder samples denoted as AM2, AM4, AM6, and AM8 respectively. Milling was interrupted for 30 min every half an hour to prevent overheating. Milling was performed in an inert argon atmosphere to prevent any oxidation. As-milled powder samples were characterized using scanning electron microscopy (SEM), X-ray diffraction (XRD), and vibratory sample magnetometry (VSM).

**Fig. 1:**
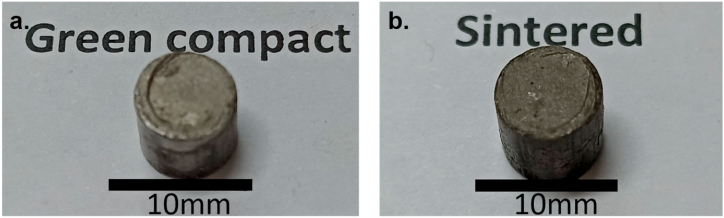
AMC samples after a. After Compaction (Green Compact) and b. after sintering

Following milling, cold compaction was carried out. Then, an axial hydraulic press was utilized at 500 MPa compaction pressure to prepare cylindrical samples of 8 mm diameter and 4 mm height, as shown in Fig. 1a. Following compaction; the samples were sintered in a vacuum furnace at 600 °C with a heating rate of 5 °C/min, hold time of 2 h, then left to cool overnight, as reflected in Fig. 1b.

After sintering, XRD, SEM, and Energy Dispersive Spectroscopy (EDS) were used for morphology and composition characterization. According to MPIF 42, 1998 standards, the synthesized composites' density was measured usingArchimedes’ method employing distilled water as the floating liquid. Weighing was done using pure water and air for the sintered samples. Micro Vickers Hardness Testing (HMV-G, Shimadzu) was used to measure hardness. The load was set to 100 g with a dwell time of 10 s. An average hardness value of 15 readings was taken.

## 3 Results and discussion

This section of the paper starts with the characterization results of the MagNPs in terms of morphology, structure, and magnetic properties. This is followed by characterization results of the as-milled powder samples (AM2, AM4, AM6, and AM8) regarding morphology, structure, and magnetic properties. Finally, the AM8 powder sample is selected for sintering and characterized in composition, morphology, and hardness. The AM8 sample was chosen for sintering and further testing since it exhibited the smallest particle size and highest magnetic properties.

### 3.1 Characterization of MagNPs

MagNPs are characterized in terms of morphology using TEM (JEOL JEM-2100) to assess particle morphology and size. Structural characterization was performed using XRD using an XRD-6100 diffractometer by Shimadzu equipped with a Cu-Kα source. Finally, magnetic properties are investigated using VSM (Lake Shore 7410) and compared to values reported in literature.

#### 3.1.1 Transmission electron microscopy (TEM) of MagNPs in suspension state

**Fig. 2:**
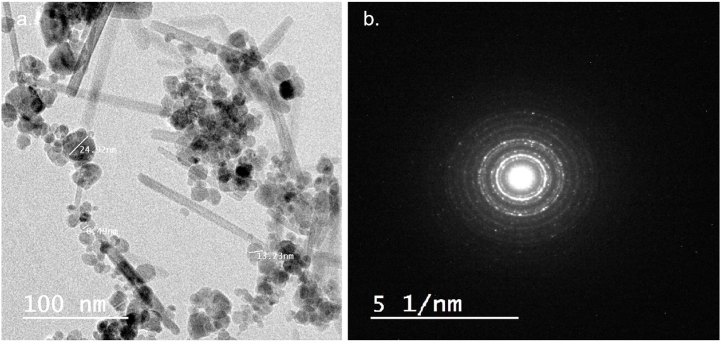
TEM analysis of the MagNPs: a. TEM micrograph at 5000× and b. The selected area diffraction (SAD) pattern.

Fig. 2a shows the TEM micrograph of the MagNPs at 50,000× magnification. The MagNPs have a spherical shape with varying sizes between 10 and 30 nm, comparable to results by Liang et al., who had spherical particles with a 12 nm diameter [24]. Results for selected area diffraction (SAD) are shown in Fig. 2b. The SAD pattern shows dotted concentric rings indicating a crystalline material.

#### 3.1.2 XRD analysis of the MagNPs

**Fig. 3:**
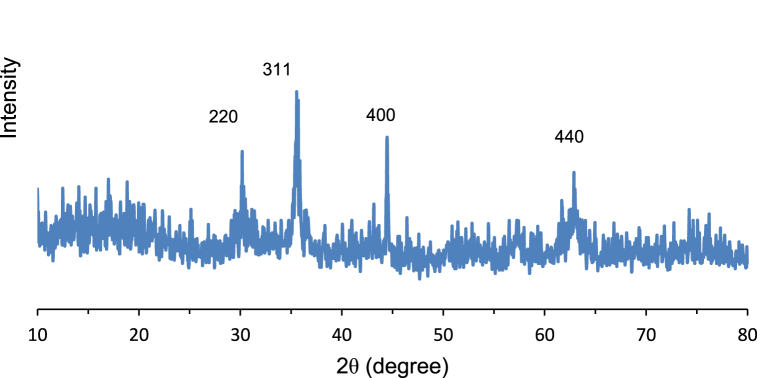
XRD analysis of the MagNPs.

The X-ray diffraction measurements of the MagNPs in Fig. 3 show peaks of magnetite (Fe_3_O_4_) with an absence of any impurities. The diffraction peaks matched with reference peaks of Fe_3_O_4_ according to JCPDS card No. 00-019-062. This is indicated by the (220), (311), (400), and (440) characteristic peaks of Fe_3_O_4,_ with the (311) peak having the highest intensity and occurring at 2θ = 35.3°.

#### 3.1.3 Vibratory sample magnetometry (VSM) of MagNPs

**Fig. 4:**
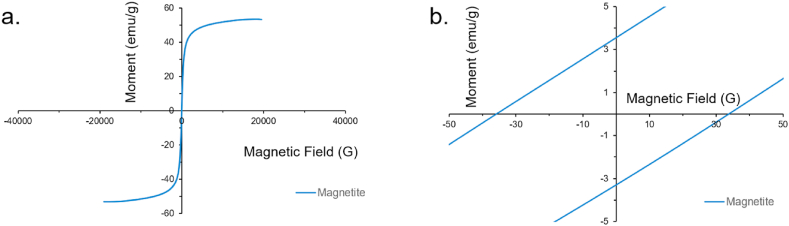
Magnetic properties investigation of the MagNPs using VSM: a. The Magnetic Saturation (Ms) of the MagNPs and b. The Magnetic Remanence (Mr) of the MagNPs.

Magnetic measurements using VSM were performed to determine the magnetic properties of the MagNPs. The magnetic saturation (M_s_) was determined to be 53.436 emu/g, as shown in Fig. 4a., indicating high magnetism as expected from magnetite. Fig. 4b shows that the MagNPs exhibited magnetic remanence (M_r_) of 3.5 emu/g. Magnetic remanence indicates how magnetic the material remains after removing the magnetic field. Our results show that the magnetic behavior of the fabricated MagNPs is similar to results reported in literature [[27], [28], [29], [30]], indicating its suitability as a magnetic reinforcer for AMCs.

### 3.2 Characterization of the as-milled powder at different time durations

With the MagNPs characterized, the different as-milled powder samples (AM2, AM4, AM6, and AM8) were characterized in terms of morphology using SEM (JEM-6500F, JEOL), in terms of structure using XRD and in terms of magnetic properties using VSM. The goal is to find the optimum milling time for enhanced magnetic and mechanical properties.

#### 3.2.1 SEM of as-milled powder for different milling times

**Fig. 5:**
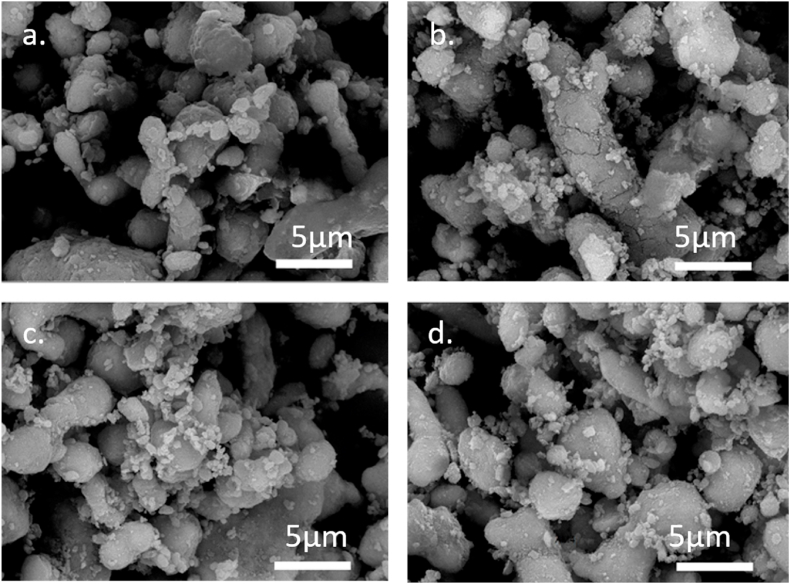
SEM of the 4 as-Milled powder compositions: a. AM2, b. AM4, c. AM6, and d. AM8. With a scale bar of 5 μm.

Throughout the SEM micrographs in Fig. 5., at 15,000× magnification, a noticeable homogenous distribution of the magnetite, brighter smaller particles, in the aluminum matrix is found. After 2 h of milling, a homogenous distribution is noticed, as shown in Fig. 5a. With increased milling time, the aluminum particles are deformed and show elongated particles with cracks along their structure, as illustrated in Fig. 5b, then they assume spherical-like shapes with diameters between 5 and 10 μm as shown in Fig. 5c and d. The magnetite particles also seem to have reduced in size, from about 500 nm platelet-like structures in Fig. 5a to more homogenous spherical shapes of about 100–200 nm after 8h milling time, as shown in Fig. 5d. This decrease in size is expected due to the longer milling time.

#### 3.2.2 XRD analysis of the as-milled powder

**Fig. 6:**
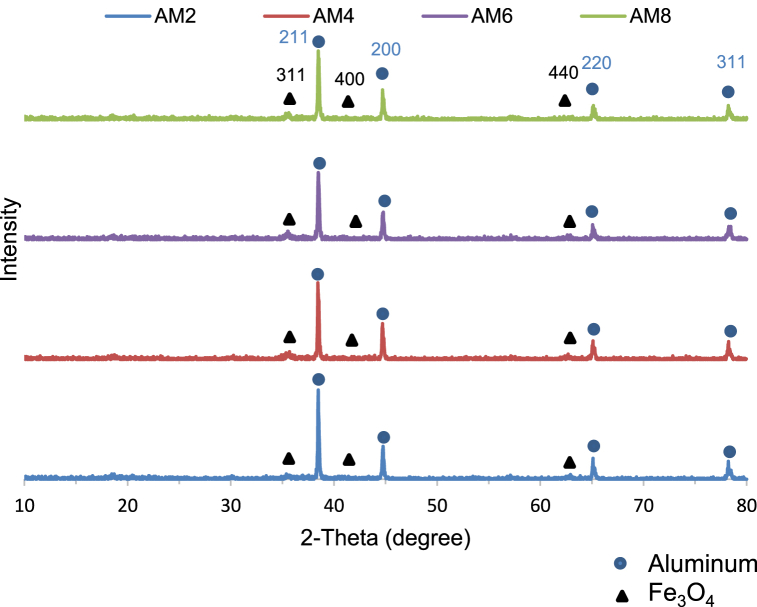
XRD analysis of the 4 as-Milled powder compositions: AM8, AM6, AM4, and AM2 arranged from top to bottom.

As-milled powder samples were characterized using XRD to determine the effect of the milling time on the crystallinity and formation of any intermetallic compounds. As shown in Fig. 6., all graphs indicate the presence of aluminum and magnetite only, without any other oxides or compounds. Accordingly, no overheating occurred even at 8 h of milling with the AM8 sample. Focusing on the AM2 and the AM8 XRD graphs, there is slight to no change in peak location, yet a decrease in aluminum peak intensities with some broadening of the peak, indicating aluminum refining. Therefore, it is safe to claim that particle size decreases as the milling time increases.

From the SEM, XRD, and VSM results, the AM8 sample showed the smallest particle size in the SEM micrographs, further confirmed by the XRD results. It also had the highest M_s_ value. Therefore, it was chosen for further characterization and hardness testing after compaction and sintering.

#### 3.2.3 Magnetic properties of the as-milled powder

**Fig. 7:**
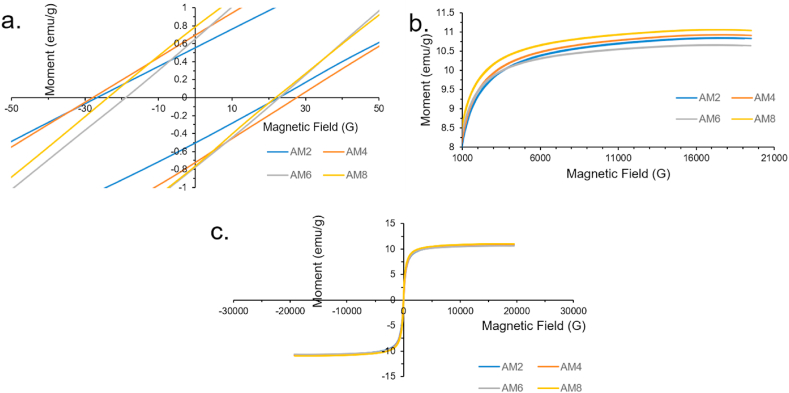
Magnetic properties investigation of the 4 As-Milled powders (a. AM2, b.AM4, c. AM6, and d. AM8) using VSM: a. VSM measurement summed up, b. the Ms of the 4 powders, and c. the Mr of the 4 powders.

With increasing milling time, the magnetic properties of the powder did not show a substantial change. All samples had a magnetic saturation (M_s_) ranging between 10.658 emu/g and 11.029 emu/g, with the AM8 showing the highest value. Results are shown in Fig. 7a and b. The M_s_ data indicate a higher magnetic affinity than values reported in literature of about 3.86 emu/g at double the magnetite percentage (20 wt%) [23]. Another study reached about 13.44 emu/g at triple the magnetite percentage (30 wt%) [19].

Results also show that magnetic remanence (M_r_) increases with increasing milling time, as indicated in Fig. 7c starting at 0.5 emu/g for the AM2 sample and reaching 0.8 emu/g for the AM8 sample. The M_r_ values reported in literature were 0.065emu/g [19] and 0.08emu/g at the same magnetite wt% [23]. Having high saturation and low remanence, with a thin and long hysteresis loop, the composite at hand qualifies as a soft magnetic material that can be used in various electromagnetic applications.

### 3.3 Characterization and morphology of the AM8 sample

The as-milled AM8 powder was further characterized before compaction and sintering using EDS to investigate its chemical composition and the distribution of magnetite and aluminum in the powder. It was then compacted and sintered, followed by SEM and EDS characterization to check for homogeneity, composition, and particle size. Finally, hardness testing was performed to check for mechanical properties enhancement.

#### 3.3.1 Compositional distribution of the as-milled AM8 powder using EDS

**Fig. 8:**
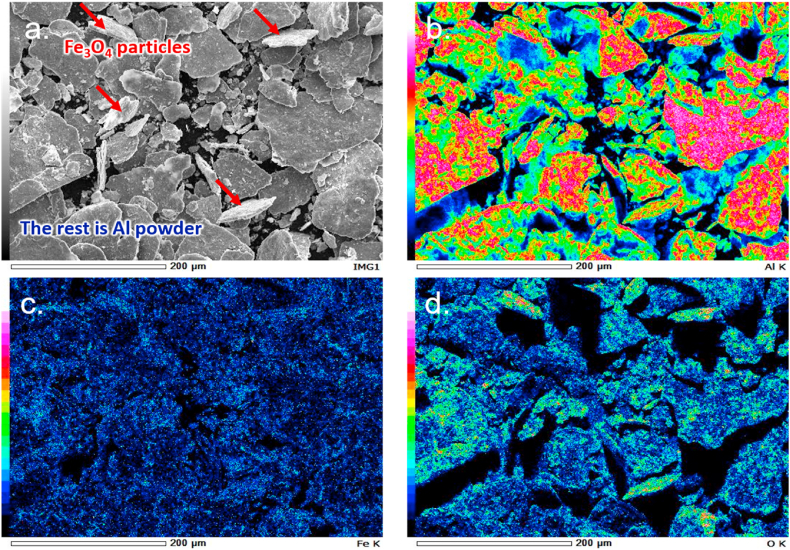
Compositional analysis of the as-Milled AM8 powder: a. SEM reference image showcasing the difference between the magnetite and aluminum, b. The aluminum concentration as detected by the EDS, c. The Iron concentration as detected by the EDS, and d. The Oxygen concentration, as detected by the EDS.

An SEM micrograph of the as-milled AM8 composite powder sample at 250× is shown in Fig. 8a. EDS micrographs clearly distinguish between the light-colored magnetite platelets and the darker, more spread aluminum platelets. The EDS micrographs also show the distribution of the different elements in the sample, as shown in Fig. 8b, c, and d. In Fig. 8b the wide distribution of the aluminum in the powder is precise, being the matrix material. Fig. 8c shows the iron concentration throughout the composite powder. Since the iron content is about 10%, iron is mainly concentrated in areas where the magnetite is. These are the slightly brighter regions in Fig. 8a.

Fig. 8d shows the oxygen presence areas, which are mostly related to the presence of magnetite and any aluminum oxides that may be present. However, comparing Fig. 8a and d, magnetite can be identified from its higher oxygen content, while other oxygen-rich zones are scarce, indicating the insignificant presence of aluminum oxides. This further confirms the XRD results in Fig. 6, where no aluminum oxides were detected in any milled powder.

#### 3.3.2 SEM results for the sintered composite AM8-s

**Fig. 9:**
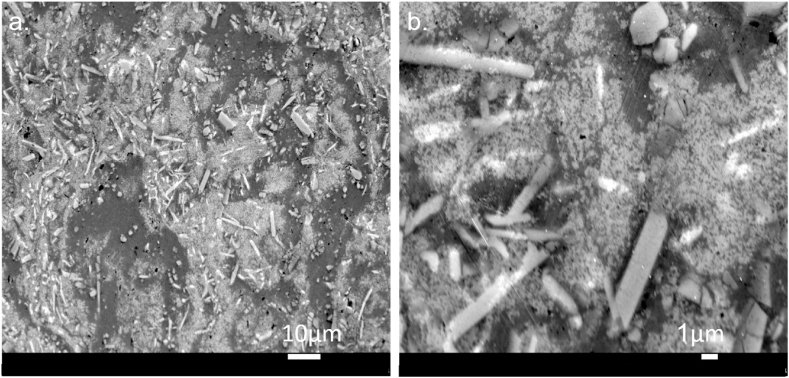
SEM of the As-Sintered AM8 at two magnifications: a. 1000× with a scale bar of 10 μm and b. 5000× with a scale bar of 1 μm.

The composite morphology was examined with SEM as shown in Fig. 9. Fig. 9a at 1000× magnification shows rod-shaped bright particles, finer spherical particles, and a darker background. Fig. 9b displays the rod-shaped structures at higher magnification (5000×). The microstructure of sintered AMCs in previous studies is composed of spherical particles with a size distribution of 5–10 μm [21,22,24]. The AM8-s in the current study also showed similar particle sizes between 10 and 30 μm. However, the rod-shaped particles are unique to this study. The rod-shaped particles are composed of magnetite due to the bright color, like the as-milled AM8 results in Fig. 8a. The reason behind the particles assuming a rod shape may be due to the compaction conditions. This study used a compaction pressure of 500 MPa, double what was used in the literature [21,22,24]. Therefore, it is hypothesized that the magnetite platelets broke into these rod-shaped particles. This could be attributed to magnetite's brittle nature, aluminum's conformity and ductility, and the high compaction pressure.

#### 3.3.3 Compositional distribution of the as-sintered AM8 sample using EDS

**Fig. 10:**
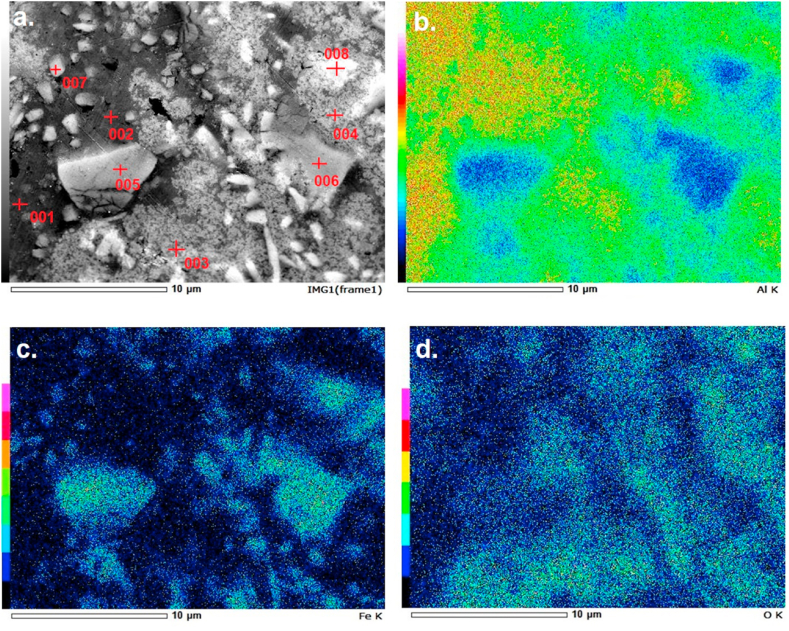
Compositional analysis of the As-Sintered AM8 composite (AM8-s): a. SEM micrograph as a reference to the EDS, b. Aluminum concentration detected by the EDS, c. Iron concentration detected by the EDS, and d. Oxygen concentration detected by the EDS.

Investigating the area shown in Fig. 10a using EDS, results indicate a wide distribution of aluminum as shown in Fig. 10b. Darker areas indicate a low concentration of aluminum and, accordingly, a high concentration of MagNPs, which can be further confirmed by Fig. 10c showing the higher distribution of iron within the investigated area. Fig. 10c further proves that the brighter rod-like particles observed in Fig. 9 correspond to magnetite. Fig. 10d., however, shows a wide distribution of oxygen throughout the whole composite indicating the formation of aluminum oxides after the sintering process.

To get more details on the percentage of the different elements in the composite, EDS was done at specific points, as shown in Fig. 10a. Results are shown in Table 1. As shown and discussed earlier, the brighter the color, the more iron content there is. Points 5, 6, and 8 show high iron concentrations of 17.58%, 24.25%, and 21.57%, respectively, coupled with percentages of aluminum above 70%. These regions correspond to aluminum iron intermetallic compounds. Dark spots, on the other hand, indicate a high aluminum percentage, like points 1 and 2 showing aluminum concentrations of more than 95%. Points 3 and 4 show a combination of high oxygen and aluminum content. These regions correspond to aluminum oxide that formed during the sintering process.

**Table 1:** The EDS analysis points and their respective compositions.

Point no.	Oxygen %	Aluminum %	Iron %
1	0.71	99.13	0.17
2	1.95	97.61	0.44
3	20.05	77.25	2.70
4	19.03	77.22	3.75
5	4.17	78.25	17.58
6	1.39	74.36	24.25
7	1.61	91.54	6.84
8	6.82	71.61	21.57

#### 3.3.4 XRD of the sintered composite

**Fig. 11:**
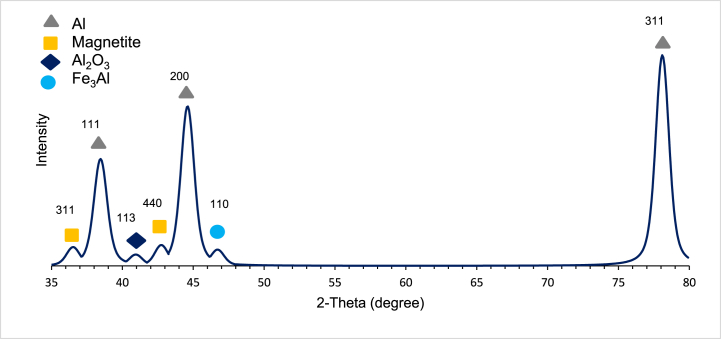
XRD analysis of the As- Sintered AM8 composite (AM8-s).

XRD analysis was necessary to validate the results determined by the EDS. After baseline subtraction, curve smoothing, and peak fitting, high-intensity peaks representing the different phases present are shown in Fig. 11. Aluminum peaks were observed being the matrix material. One magnetite peak indicated the reinforcing material's presence even after sintering. Aluminum oxide (Al_2_O_3_) is also present after sintering, as indicated by the peak at 2θ = 42.73°, indicating the oxidation of some of the matrix aluminum. Intermetallic Fe_3_Al compound was also detected in the analysis as indicated by the peak at 2θ = 46.69°. The formation of the Intermetallic Fe_3_Al compound was reported in literature after the sintering of AMCs reinforced with magnetite [31]. These results agree with the EDS results that showed the presence of aluminum oxide and intermetallic aluminum iron. The formation of Al_2_O_3_ and Fe_3_Al was also reported in the literature after compaction and sintering ball-milled Aluminium-15% magnetite powders at 600 °C [32].

### 3.4 Density measurements of the AM8-s sample

The composite's theoretical density calculated using the mixture rule was 2.8358 g/cm^3^. Then, using the Archimedes principle, as shown in eq. (1), the density of the sample was obtained. Three samples showed an average relative density of 95% (2.6940 g/cm^3^) within a standard deviation of ±1.5%. Afterward, the porosity was calculated using eq. (2) to be about 5%.

(1) $$Realtive\;density=\frac{weight\;in\;air}{loss\;of\;weight\;in\;water}=\frac{W1}{W1-W2}=\frac{d1}{d1-d2}$$

(2) $$Porosity=1-\frac{Actual\;density}{Theoritical\;density}=1-Realtive\;density$$

### 3.5 Mechanical properties of composite

The results of the Vickers hardness test for the As-sintered AM8 (AM8-s) sample revealed a hardness value of 81 Hv. A significant increase of 270% was obtained when compared to an unreinforced aluminium hardness value of 30 Hv [13].

This performance is also noteworthy when compared to AMCs reported in the literature, which include 25% wt% magnetite (Al-Mag 25% wt%) [20], are sintered by microwave sintering, and have a hardness value of 45Hv [33].

**Fig. 12:**
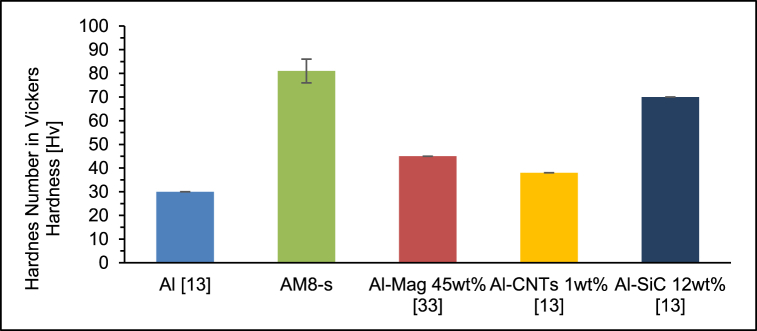
Hardness values of different AMCs in the literature compared to this study.

The hardness value in this study is substantially higher than that of AMCs with different reinforcing elements, such as carbides or Carbon Nano Tubes (CNTs). For example, AMCs containing 12 wt% SiC (Al–SiC 12 wt%) had a hardness value of 70Hv, but AMCs containing 1 wt% CNTs (Al-CNTs 1 wt%) had a hardness value of 38Hv [13]. Fig. 12 summarizes and compares the hardness values mentioned earlier.

The present AMC's increased hardness and mechanical characteristics can be linked to the tiny grain size of the aluminum powder and MagNPs and the milling time. As the milling time increases, the grain size decreases, and grain boundaries increase, blocking the movement of dislocations and producing a more rigid, more robust material [34]. With a prolonged milling time of 8 h, compared to the 2 h milling time used in literature [13,[20], [21], [22], [23], [24],^33^], there is a more significant reduction in grain size leading to a significant increase in hardness.

The results of this study must be viewed in light of some limitations. First, only the milling time and its effect on microstructure, magnetic and mechanical properties were investigated as the variable parameter. Other ball milling parameters were kept constant. However, a prolonged milling time of 8 h resulted in superior properties compared to those reported in literature. Furthermore, the current study shows unprecedented results, like rod-shaped magnetite particles. Accordingly, future studies investigating the effect of varying parameters such as the compaction pressure, ball-to-powder ratio, or a further extended milling time on the morphology of the resulting microstructure could lead to further enhancement in properties.

## 4 Conclusion

This study investigated the effect of ball milling time on fabricating AMCs reinforced with MagNPs with improved magnetic and mechanical properties. From the SEM micrographs and VSM measurements, the AM8 sample milled for 8 h showed the most promising results, with the finest grains and a high magnetic saturation of 11.029 emu/g.

After sintering the AM8 sample, SEM investigation revealed a homogeneous distribution of rod-shaped magnetite structures in aluminum. These rod-shaped structures might have developed due to the compaction pressure and the brittle nature of magnetite.

The Vickers hardness test showed a significant improvement in the hardness of this AMC (81 Hv) with a 270% increase over unreinforced aluminum. The hardness value is also 180% higher than that reported in the literature for AMCs with 2.5 times the magnetite concentration. Such enhanced mechanical performance will allow for broader use of aluminum in structural, abrasive, and wear applications. Furthermore, our investigation underlines the potential for ball-milling as a facile procedure for synthesizing advanced functional materials.

## Author contribution statement

Ayman Elsayed, Ahmed Shash, Mohamed Mitwally, Per Löthman, Hisham Mattar: Conceived and designed the experiments; Performed the experiments; Analyzed and interpreted the data; Contributed reagents, materials, analysis tools or data; Wrote the paper.

## Data availability statement

The authors confirm that the data supporting the study's conclusions are included in the article.

## Declaration of competing interest

The authors declare that they have no known competing financial interests or personal relationships that could have appeared to influence the work reported in this paper.
